# Multilevel and Multidimensional Features of Socioeconomic Status and Cognitive Function in Older Adulthood: Findings from the IGNITE Study

**DOI:** 10.1002/alz70861_108795

**Published:** 2025-12-23

**Authors:** Jermon Drake, Lu Wan, Peter Gianaros, Patricio Solis‐Urra, Haiqing Huang, Rebecca Reed, Charles Hillman, Eric D Vidoni, Jeffrey M. Burns, Arthur F. Kramer, Edward McAuley, George Grove, Chaeryon Kang, Kirk I. Erickson, Lauren Oberlin

**Affiliations:** ^1^ University of Pittsburgh, Pittsburgh, PA USA; ^2^ AdventHealth Neuroscience Institute, Orlando, FL USA; ^3^ AdventHealth Research Institute, Orlando, FL USA; ^4^ AdventHealth Research Institute, Neuroscience, Orlando, FL USA; ^5^ Northeastern University, Boston, MA USA; ^6^ University of Kansas Medical Center, Alzheimer's Disease Research Center, Fairway, KS USA; ^7^ University of Kansas Alzheimer's Disease Research Center, Kansas City, KS USA; ^8^ Center for Cognitive & Brain Health, Northeastern University, Boston, MA USA; ^9^ University of Illinois, Urbana, IL USA

## Abstract

**Background:**

Lower socioeconomic status (SES) is associated with increased risk of cognitive decline and dementia. However, due to measurement limitations that often oversimplify the multidimensional construct of SES, fundamental questions regarding the relationship between socioeconomic disadvantage and cognition remain. We examined multiple SES characteristics at the individual and area‐level simultaneously in association with diverse cognitive processes in a large, community‐based older adult sample.

**Method:**

We conducted a cross‐sectional analysis of 648 cognitively unimpaired older adults (Mean age = 69.88 + 3.75 years) enrolled in a multi‐site, randomized clinical trial: Investigating Gains in Neurocognition in an Intervention Trial of Exercise (IGNITE). Participants completed a comprehensive cognitive assessment that included tests of episodic memory, executive function, processing speed, working memory, and visuospatial abilities. Multiple dimensions of SES were obtained including area‐level (Area Deprivation Index), subjective (US Ladder from MacArthur Socioeconomic Status Index), and objective SES. We generated a novel data‐driven measure of objective SES from measures of income, savings, debt‐adjusted savings, and financial stability, and examined associations of SES indicators with cognition in multiple linear regression models that controlled for age, sex, race/ethnicity, and study site.

**Result:**

Lower objective SES was associated with poorer performance on all cognitive domains: episodic memory (β = 0.115, *p* = 0.003), executive function (β = 0.210, *p* < 0.001), processing speed (β = 0.224, *p* < 0.001), working memory (β = 0.199, *p* < 0.001), and visuospatial abilities (β = 0.164, *p* < 0.001). Subjective SES was associated with poorer performance on all cognitive domains except episodic memory. Area‐level SES was not associated with any cognitive domain (ps > 0.05). When examining all SES indicators simultaneously, only the objective SES composite remained significantly associated with cognitive function. In secondary models, the objective SES composite score explained additional variance beyond education, income, and their standardized average.

**Conclusion:**

Objective SES showed the broadest and most robust associations with cognitive function relative to subjective and area‐level indicators among cognitively normal older adults. Future studies may benefit from examining multiple financial indicators to better detect the extent and magnitude of cognitive deficits related to socioeconomic disadvantage in older adulthood.

